# Experience with early commencement of immunoglobulin treatment in score-identified high risk septic patients postoperatively after congenital heart surgery

**DOI:** 10.1186/1749-8090-10-S1-A280

**Published:** 2015-12-16

**Authors:** Zeena Makhija, Amit Kumar, Mahesh Bhatt

**Affiliations:** 1 Department of Congenital Cardiothoracic surgery, Shree Krishna Hospital, Gujarat, India; 2 Department of Pediatric Cardiac Intensive Care, Shree Krishna Hospital, Gujarat, India; 3 Department of Pediatric Cardiology, Shree Krishna Hospital, Gujarat, India

## Background/Introduction

There is still considerable controversy in literature with regards to the immunoglobulin therapy and its role in reduction of sepsis in patients undergoing congenital heart surgery.

## Aims/Objectives

The purpose of this study was to evaluate the efficacy of a polyclonal intravenous immunoglobulin preparation (IVI g) (Globucel) in the treatment of a group of patients affected by sepsis after undergoing repair of congenital heart defects in reducing overall mortality (primary endpoint). Secondary endpoints analysed included improvement in the severity score, hemodynamic variables, Arterial Blood Gas parameters Procalcitonin levels, leukocytes, platelets and coagulation profile.

## Method

This was a retrospective study. 14 patients developed sepsis in the postoperative period after congenital cardiac surgery and were admitted to the cardiovascular intensive care unit from Feb 2014 to May 2015.

All 14 patients (IVIg group) received immunoglobulins in addition to the conventional therapy. The control group consisted of a historical cohort of 14 cardiac surgical patients (age and gender matched) with comparable congenital heart disease and sepsis severity (control group) treated only with conventional therapy.

## Results

Of the 28 patients, 4 patients had septic shock (1 from the IVI g group and 3 from the control group). Sepsis was present in 11 patients (5 from the IVI g group and 6 from the control group) and severe sepsis in 13 patients (8 from the IVI g group and 5 from the control group). The overall mortality rate was 64% without significant difference between the two groups (57% IVI g group v 71% control group, p: 0.46). Among the 13 patients affected by severe sepsis, those from the control group had a mortality rate significantly higher in the control group 4/5 [80%]) as compared to the IVI g group (2/8 [25%]) (p < 0.01). The 90-day survival rate was significantly higher in the IVI g group than in the control group (log-rank test, p < 0.05). No significant differences were found between study groups in APACHE II scores.

## Discussion/Conclusion

Though the overall mortality was not significantly reduced, in the subgroup of patients with severe sepsis, the administration of polyclonal immunoglobulins improved the survival rate significantly.

